# Relevance of miR-21 in regulation of tumor suppressor gene PTEN in human cervical cancer cells

**DOI:** 10.1186/s12885-016-2231-3

**Published:** 2016-03-14

**Authors:** Oscar Peralta-Zaragoza, Jessica Deas, Angélica Meneses-Acosta, Faustino De la O-Gómez, Gloria Fernández-Tilapa, Claudia Gómez-Cerón, Odelia Benítez-Boijseauneau, Ana Burguete-García, Kirvis Torres-Poveda, Victor Hugo Bermúdez-Morales, Vicente Madrid-Marina, Mauricio Rodríguez-Dorantes, Alfredo Hidalgo-Miranda, Carlos Pérez-Plasencia

**Affiliations:** Direction of Chronic Infections and Cancer, Research Center in Infection Diseases, National Institute of Public Health, Av. Universidad No. 655, Cerrada los Pinos y Caminera, Colonia Santa María Ahuacatitlán, Cuernavaca, Morelos, México, 62100 Mexico; Clinical Research Laboratory, Academic Unit of Biological Chemical Sciences, Guerrero Autonomous University, Avenida Lázaro Cárdenas S/N, Col. Haciendita, Chilpancingo, Guerrero, México, 39070 Mexico; Pharmaceutical Biotechnology Laboratory, Faculty of Pharmacy, Autonomous University of Morelos State, Avenida Universidad No. 1001, Cuernavaca, Morelos, México, 62010 Mexico; National Institute of Genomic Medicine, Periférico Sur No. 4809, Col. Arenal Tepepan, Delegación Tlalpan, México, D.F. C.P. 14610 Mexico; Oncogenomics Laboratory, National Cancer Institute of Mexico, Tlalpan, Av. San Fernando No. 22, Colonia Sección XVI, Delegación Tlalpan, Distrito Federal, México, 14080 Mexico; Biomedicine Unit, FES-Iztacala UNAM, Av. De los Barrios S/N. Colonia Los Reyes Iztacala, Tlalnepantla de Baz, Estado de México, 54090 Mexico; CONACyT Research Fellow-Instituto Nacional de Salud Pública (INSP), Cuernavaca, Morelos Mexico

**Keywords:** Cervical cancer, microRNAs, miR-21, PTEN, siRNAs

## Abstract

**Background:**

Expression of the microRNA miR-21 has been found to be altered in almost all types of cancers and it has been classified as an oncogenic microRNA or oncomir. Due to the critical functions of its target proteins in various signaling pathways, miR-21 is an attractive target for genetic and pharmacological modulation in various cancers. Cervical cancer is the second most common cause of death from cancer in women worldwide and persistent HPV infection is the main etiologic agent. This malignancy merits special attention for the development of new treatment strategies. In the present study we analyze the role of miR-21 in cervical cancer cells.

**Methods:**

To identify the downstream cellular target genes of upstream miR-21, we silenced endogenous miR-21 expression in a cervical intraepithelial neoplasia-derived cell lines using siRNAs. The effect of miR-21 on gene expression was assessed in cervical cancer cells transfected with the siRNA expression plasmid pSIMIR21. We identified the tumor suppressor gene PTEN as a target of miR-21 and determined the mechanism of its regulation throughout reporter construct plasmids. Using this model, we analyzed the expression of miR-21 and PTEN as well as functional effects such as autophagy and apoptosis induction.

**Results:**

In SiHa cells, there was an inverse correlation between miR-21 expression and PTEN mRNA level as well as PTEN protein expression in cervical cancer cells. Transfection with the pSIMIR21 plasmid increased luciferase reporter activity in construct plasmids containing the PTEN-3′-UTR microRNA response elements MRE21-1 and MRE21-2. The role of miR-21 in cell proliferation was also analyzed in SiHa and HeLa cells transfected with the pSIMIR21 plasmid, and tumor cells exhibited markedly reduced cell proliferation along with autophagy and apoptosis induction.

**Conclusions:**

We conclude that miR-21 post-transcriptionally down-regulates the expression of PTEN to promote cell proliferation and cervical cancer cell survival. Therefore, it may be a potential therapeutic target in gene therapy for cervical cancer.

## Background

MicroRNAs are a recently discovered family of genes encoding small RNA molecules of 19–25 nucleotides in length which bind through partial sequence homology to the 3′-untranslated regions (3′-UTRs) of mRNA from cell target genes, induce repression of translation and, as a result, play key roles in the regulation of gene expression and of the dynamics of development in a great variety of eukaryotic organisms [1]. *Homo sapiens* miR-21 (hsa-miR-21) is one of first microRNAs detected in the human genome and to date is the major oncomir known to be up-regulated in all types of human cancer including glioblastoma multiforme [2], breast [3], lung [4], esophageal [5], gastrointestinal [6], hepatocellular [7], cholangiocarcinoma [8], pancreatic [9], prostate [10], bladder [11], ovarian [12], NK-cell lymphoma [13], laryngeal carcinoma [14] and tongue squamous cell carcinoma [15]. Therefore, much research has been conducted to determine its physiological and pathophysiologycal functions during embryonic development and cell proliferation, differentiation and death [16–19]. Recently, an integral role for miR-21 in tumor pathogenesis has emerged, with extensive studies indicating that miR-21 is involved in all known cancer-related processes including tumorigenesis, progression and metastasis [19–22]. Furthermore, the level of miR-21 expression is significantly associated with clinical-pathological factors and the prognosis of cancer patients, suggesting that it could be utilized as a diagnostic and prognostic marker in human malignancy [23–28].

Currently, there are few microRNAs whose physiologic function has been elucidated in vivo and whose gene targets are known. Among these is miR-21, located at chromosome 17q23.2 locus, which codes for pri-miR-21 located within the intronic region of the protein-coding gene TMEM49 [25]. Inhibition of miR-21 can induce cell cycle arrest and increase chemosensitivity to anticancer agents, providing evidence that miR-21 may function as an oncogene in various human cancers [5, 7, 9, 19, 27]. Recently, several significant miR-21 targets associated with malignancy have been identified by different groups: Phosphatase and tensin homologue deleted on chromosome ten (PTEN) [28], programmed cell death 4 protein (PDCD4) [29], reversion-inducing-cysteine-rich protein with kazal motifs (RECK) [19], maspin [30], tropomyosin 1 (TPM1) [31], heterogeneous nuclear ribonucleoprotein K (HNRPK) and TAp63 [27]. In addition, previous studies have reported that miR-21 expression levels are significantly higher in tumor cervical samples compared with their normal tissue counterparts [32–34]. However, the functional activity of miR-21 in cervical cancer cells remains largely unknown, and thus far, few miR-21 gene targets in cervical cells have been reported.

Cervical cancer is the second most common malignancy affecting women worldwide, with approximately 500,000 new cases diagnosed and 280,000 deaths occurring each year. The highest incidences occur in the developing world, where, in most countries, cervical cancer is the leading cause of cancer mortality in women [35]. Although the relationship between persistent high-risk HPV infection and cervical cancer development has been well documented in clinical, epidemiological, molecular and functional studies [36], the detailed regulatory network of events leading from HPV infection to tumor development has yet to be elucidated. An event that occurs in HPV-associated carcinogenesis during HPV DNA integration is a global perturbation of cellular gene expression, mainly by the HPV E6 and E7 oncogene expression [37–39]. Recent evidence suggests a relationship between HPV E6 and E7 oncogene expression and disruption of cellular microRNA expression. Many cellular transcription factors, including AP-1, c-Myc, E2F, NF-kB, pRb, and p53, have been determined to regulate the transcription of microRNAs [40]. Therefore, it is plausible that HPV infection causes aberrant cellular gene expression including disruption of microRNA expression.

In the present study, SiHa and HeLa cells, which are human cervical cancer cells infected with HPV16 and HPV18, respectively, were used as a cervical cancer model to investigate whether siRNA-mediated gene silencing specific to miR-21 expressed in plasmids, could be used to silence miR-21. We determined whether these siRNAs could alter the expression of the tumor suppressor gene PTEN, which has been reported as a miR-21 target gene in other malignancies. In addition, we evaluated the biological effects of siRNAs targeting miR-21 in tumor cells. To this end, we generated siRNA expression plasmids for miR-21, which have nucleotide complementarity to the gene coding for pre-miR-21. We found that these siRNAs could induce silencing of miR-21. Furthermore, we found that siRNAs against miR-21 induced the reestablishment of PTEN gene and protein expression, as well as reestablishment of its biological effects on cell proliferation. Our results indicate that SiHa and HeLa cell death occurred by autophagy and apoptosis, the latter through caspase-3/7 activity, in response to the silencing of miR-21. To describe the molecular mechanism of PTEN gene regulation by miR-21 and test its potential trans-regulatory abilities, we investigated the effect of miR-21 on the PTEN 3′-UTR regulatory region in cervical tumor cells. We found that miR-21 can trans-regulate the PTEN 3′-UTR regulatory region. This effect is the result of miR-21’s interaction with a specific MRE (microRNA response element) recognition sequence located in position 1925 to 1956 nt (denominated MRE21-2) in the PTEN 3′-UTR. Taken together, these findings demonstrate that siRNAs directed against miR-21 are excellent molecular tools to inhibit this microRNA’s expression and activities in a targeted manner to induce reestablishment of target cell gene expression, which has relevant biological effects on tumor cell progression.

## Methods

### Cell lines and culture conditions

Human cervical cancer cells transformed with HPV16 (SiHa cells), and HPV18 (HeLa cells) were obtained from the American Type Culture Collection (ATCC). The cell line was cultured in Dulbecco’s modified Eagle’s medium (DMEM) (Invitrogen, Carlsbad, CA) supplemented with 10 % fetal bovine serum (FBS), penicillin/streptomycin (50 μg/ml), 2 mM L-glutamine, 250 ng/ml fungizone, and maintained at 37 °C in 5 % CO_2_. The total RNA isolation was carried out with TriPure isolation reagent (Roche, Indianapolis, IN) for the end-point RT-PCR and real time RT-qPCR assays. The cellular protein isolation was performed and protein concentration was determined by the BCA protein kit (Pierce, Rockford, IL.) for the Western Blot assays. The cells were attached on a slide for epifluorescence microscopy assays or fixed in ethanol for the flow cytometry assays. In addition, the cells which were used in transfection assays were analyzed for the luciferase activity assays.

### siRNA expression plasmids for human microrna miR-21

DNA inserts encoding siRNAs specific for human microRNA hsa-mir-21 [miRBase: MI0000077] were designed using software from Applied Biosystems-Ambion [41] and were cloned in *Apa I* and *Eco RI* restriction sites in the pSilencer1.0-U6 siRNA expression plasmid (Applied Biosystems, Foster, CA), which contains the U6 RNA Pol-III promoter to generate small RNA transcripts, to generate the pSIMIR21 plasmid. The DNA insert was generated using the sense 5′-CAC-CAG-TCG-ATG-GGC-TGT-CTT-CAA-GAG-AGA-CAG-CCC-ATC-GAC-TGG-TGT-TTT-TT-3′ and antisense 5′-AAT-TAA-AAA-ACA-CCA-GTC-GAT-GGG-CTG-TCT-CTC-TTG-AAG-ACA-GCC-CAT-CGA-CTG-GTG-GGC-C-3′ primers. The primers were aligned using annealing buffer (300 mM HEPES pH 7.4, 100 mM potassium acetate, 2 mM magnesium acetate) at a ratio of 100 mM and incubated at 95 °C for 5 min and 37 °C for 1 h. To address the possibility of homologous sequences with other human genes, the siRNAs-encoding sequences were analyzed with Blast. The integrity of all plasmid constructs was verified by DNA sequencing in Genetic Analyser 3500xl equipment (Applied Biosystems, Foster, CA).

### Transfection assays with siRNA expression plasmids

SiHa and HeLa cells were transiently transfected with pSIMIR21 plasmid to silence miR-21, using Fugene HD transfection reagent (Promega, Madison, WI) according to the manufacturer’s instructions. Briefly, one day before the transfection assay, the cells were plated at a density of 1X10^5^ cells per well in a six-well plate containing 2 ml of DMEM with 10 % FBS and penicillin/streptomycin. At the time of transfection, the plasmids and Fugene reagent were diluted in DMEM and incubated for 20 min at room temperature. The plasmid DNA concentration and Fugene reagent were normalized by transfection with pGFP plasmid and all assays were carried out with 0, 3 and 5 μg of plasmids. SiHa and HeLa cells were incubated with plasmids and Fugene for 4 h, rinsed and replenished with DMEM containing 10 % FBS. The plasmids were isolated with PureYield plasmid midiprep system (Promega, Madison, WI) and integrity was verified by DNA sequencing. After 48 h of transfection the cells were harvested and RNA isolation was carried out for semiquantitative end-point RT-PCR as well as for quantitative real-time RT-PCR assays. Cellular protein isolation was performed by Western Blot assays. After transfection, cells were used for epifluorescence microscopy as well as flow cytometry and evaluation of reporter plasmid activity and caspases-3/7 activity. Transfection assays were repeated at least four times independently.

### Cellular viability assays

Cellular viability was measured using [3-(4,5-dimethylthiazol-2-yl)-5-(3-carboxymethoxyphenyl)-2-(4-sulfophenyl)-2H-tetrazolium] inner salt MTS assay (Promega, Madison WI), which is a colorimetric method for determining the number of viable cells in a proliferation or cytotoxicity assay. Briefly, a total of 2X10^4^ SiHa cells per well were plated in a 96-well plate. After 24 h of plating, 20 μl of MTS reagent was added into each well containing the untreated cells and cells transfected with pSIMIR21 plasmid in 100 μl DMEM, and these were incubated at 37 °C for 4 h. MTS tetrazolium compound salt reagent is bioreduced by living cells into a colored formazan product that is soluble in tissue culture medium. After incubation, the absorbance values were measured at 490 nm in an automatic microplate reader. Cellular viability rate was calculated as the percentage of MTS adsorption as follows: % survival = (mean experimental absorbance/mean control absorbance) X 100. Each assay was carried out three separate times.

### Semiquantitative end-point RT-PCR analysis

Transfected SiHa cells were harvested and processed for total RNA isolation using TriPure isolation reagent (Roche, Indianapolis, IN) according to the manufacturer’s protocol. Briefly, cells were washed with 1X PBS and 1 ml TriPure was added. 200 μl chloroform was added and the cells were centrifuged. The aqueous phase was separated and the RNA was precipitated with isopropanol. The RNA was dissolved in DEPC-water and the concentration was measured. The mRNA was obtained using oligo dT dT15-18 (Promega, Madison, WI) and cDNA synthesis was performed by incubation with M-MLV reverse transcriptase (Promega, Madison, WI) at 37 °C during 1 h. Homo sapiens PTEN gene expression [NCBI: NM_000314] was measured by semiquantitative end-point RT-PCR using the sense 5′-GGG-AAG-ACA-AGT-TCA-TGT-AC-3′ and antisense 5′-AGT-ATC-GGT-TGG-CTT-TGT-C-3′ primers which were generated using the GeneFisher2-interactive PCR primer design software [42]. The PCR reaction amplification conditions were 95 °C for 10 min, 95 °C for 1 min, 55 °C for 30 s and 72 °C for 1 min for 35 cycles followed by 72 °C for 10 min. A 309 bp DNA fragment was obtained for PTEN gene. The glyceraldehyde-3-phosphate dehydrogenase (GADPH) housekeeping gene was used as a control using sense 5′-CAA-CAG-CCT-CAA-GAT-CAT-C-3′ and antisense 5′-ACC-AGG-AAA-TGA-GCT-TGA-C-3′ primers. The PCR reaction amplification conditions were 95 °C for 10 min, 94 °C for 1 min, 54 °C for 1 min and 72 °C for 1 min for 35 cycles followed by 72 °C for 10 min. A 520 bp DNA fragment was obtained. For each PCR reaction, 1 μg cDNA, 2.5 mM dNTPs, 20 pM each primer and 0.5 U Taq (Promega, Madison WI) were used in a 25 μl volume reaction. To ensure that amplification remained within the linear range, 1:5 serial dilutions of cDNA were made.

### Quantitative real time RT-PCR analysis

Total RNA isolation from SiHa cells transfected as previously described was carried out with TriPure isolation reagent (Roche, Indianapolis, IN). The cDNA synthesis was performed by incubation with 100 ng RNA, 1X RT buffer, 0.25 mM each dNTPs, 0.25 U/μl RNase-OUT inhibitor and 3.33 U/μl M-MLV reverse transcriptase, in a one step 7.5 μl volume reaction. The reaction was incubated in a 384 well plate at 37 °C during 30 min in Applied Biosystems 7900 Real-Time PCR Instrument. For analysis of miR-21 expression, real time RT-qPCR analyses were performed using TaqMan pri-miRNA assays (Applied Biosystems, Foster, CA) according to the manual. Ct values were analyzed to determine the statistical significance of miR-21 expression in SiHa cells transfected or non-transfected with pSIMIR21 expression plasmid. Relative expression was calculated using the 2^-∆∆Ct^ method and normalized to the expression of RNU6 (Applied Biosystems, Foster, CA) [43, 44]. The reaction was incubated in a 384 well plate in Applied Biosystems 7900 Real-Time PCR Instrument. All RT-qPCR were performed in triplicate.

### Western blot assays

Forty eight hours after transfection assays, SiHa cells were harvested and protein was isolated for Western Blot assays. Briefly, the cells were washed with 1X PBS and incubated for 30 minites at 4 °C with lysis buffer containing 50 mM Tris–HCl, 150 mM NaCl, 0.5 % SDS, 1 % NP40, 0.5 mM AEBSF, 10 μg/μl antipain, 10 μg/μl aprotinin, 10 μg/μl khymostatin, 10 μg/μl leupeptin, 10 μg/μl pepstatin, 1 mM EDTA, 100 mM PMSF and 0.5 mM DTT (Sigma-Aldrich, NJ). The lysates were centrifuged at 11,000 rpm for 15 min. Total proteins from supernatants were determined using the BCA kit (Pierce, Rockford, IL). 50 μg of proteins were electrophoresed on 12 % SDS-PAGE, transferred into nitrocellulose membranes and incubated for antibodies detection. Biotinilated and pre-stained molecular weight marker was included. IgG mouse monoclonal antibody sc-7974-HRP was used to detect human PTEN protein. Human beta-actin protein was detected using IgG polyclonal antibody sc-1616-HRP (Santa Cruz, Biotechnology, Santa Cruz, CA). After the peroxidase coupled secondary goat antibody mouse anti-IgG was added, bound antibodies and protein were detected by enhanced chemiluminescence using the renaissance Western Blot kit (Pierce, Rockford, IL). The membranes were subjected to autoradiography with an intensifier screen.

### Reporter plasmids and luciferase activity assays

SiHa cells were transiently transfected with pMRE21PtenLuc1 and pMRE21PtenLuc2 reporter plasmids, which contain cloned the MRE21-1 (microRNA response elements for miR-21 of 1628 to 1649 nt) and MRE21-2 (of 1925 to 1955 nt) of PTEN-3′-UTR regulatory region. The information was generated from nucleotide sequence database for PTEN [GeneBank: NM_000314.4] and for hsa-miR-21 [GeneBank: MI0000077]. The MRE21-1 and MRE21-2 were cloned in *Spe I* and *Hind III* restriction sites of pMIR-Report-Luciferase reporter vector (Life Technologies, NY), which contains a firefly luciferase reporter gene under the control of a CMV promoter/termination system. The design of construct plasmids was carried out in target scan human prediction of microRNA targets software [45]. The co-transfection assays were performed with pSIMIR21 plasmid which expresses siRNAs for miR-21. The pMRE21PtenLuc1 plasmid was generated for cloning of PCR product of 387 bp DNA fragment using the sense 5′-GAC-TGA-TCA-CTT-TCC-CGT-TTT-ATT-CC-3′ and antisense 5′-CCC-AAG-CTT-AAT-GCG-CAA-ACA-ACA-AGC-3′ primers. The pMRE21PtenLuc2 plasmid was generated for cloning of PCR product of 364 bp DNA fragment using the sense 5′-GAC-TAG-TTT-GGC-TAA-GAG-AGG-TTT-CC-3′ and antisense 5′-CCC-AAG-CTT-TTG-TTG-CTG-TGT-TTC-TTA-CC-3′ primers. The plasmids were isolated by PureYield plasmid midiprep system (Promega, Madison, WI) and the integrity was verified by DNA sequencing. SiHa cells were transfected using Fugene reagent (Promega, Madison, WI) according to the manufacturer’s instructions as mentioned above. The beta-galactosidase activity was not affected by pMRE21PtenLuc plasmids in SiHa cells, therefore the luciferase activity in all assays were normalized using the pMIR-Report-Luciferase empty vector and pMIR-Report-beta-gal reporter plasmids. All transfection assays were performed with 5 μg of plasmid DNA. SiHa cells were incubated with Fugene reagent for 4 h and 48 h after transfection, cells were washed with 1X PBS and were harvested and lysed with 100 ml cold lysis buffer (20 mM Tris–HCl pH 7.4, 10 mM NaCl, 10 mM KCl, 3 mM MgCl_2_, 0.5 % Triton X-100. 0.5 % Nonidet P40). The cellular extracts were collected by centrifugation. 50 μg of total proteins were used to determine luciferase activity. Luciferase activity was measured and normalized using the Dual-Glo luciferase assay system (Promega, Madison WI) in Glomax Multidetection equipment according to the manufacturer’s instructions. The luminescence was calculated to normalize results with respect to pMIR-Report-Luciferase empty vector and the efficiency of transfection. All transfections and co-transfections were repeated at least three times independently.

### Flow cytometry assays

Transfected SiHa cells were harvested, centrifuged, fixed in cold 70 % ethanol and stored at −20 °C. Prior to analysis, the ethanol was removed and cells were incubated at room temperature for 10 min in 1 ml buffer A (1 mg/ml citric acid, 0.1 % Nonidet P40, 1.16 mg/ml spermine tetrahydrochloride, 60.5 μg/ml trizma hydrochloride pH 7.6) containing 30 μg/ml porcine pancreatic trypsin. Next, the SiHa cells were incubated at room temperature for 10 min with 1 ml of 30 μg/ml trypsinogen and 100 μg/ml ribonuclease A dissolved in buffer A. Then, SiHa cells were incubated at 4 °C for 10 min in 1 ml of 500 μg/ml propidium iodide and 1.16 mg/ml spermine tetrahydrochloride dissolved in buffer A. During each incubation, cells were vortexed intermittently every 2 min. Approximately 10,000 nuclei were processed in FACS Sort Becton Dickinson (Ar laser, 488 nm and 620 nm excitation and emission wavelengths, respectively) and results were analyzed with ModFit LT (Verity) software. Instrument settings were fixed using non-transfected SiHa cells.

### Epifluorescence microscopy with acridine orange (AO) and propidium iodide (PI) staining

This technique is based on a double staining of the cell and observation of the nucleus. AO is permeable into all cells while PI only permeates cells where membrane is compromised. The state of the nucleus is then analyzed. In the case of programmed cell death, specifically apoptosis, the cell nucleus is fragmented and is observed as green in the early apoptosis phase and red in the late apoptosis phase. During necrosis and cell death, the nucleus is stained red but the morphology is similar to the viable cells (stained green). Attached samples of SiHa and HeLa cells on a slide were harvested at 48 h after transfection and stained with 5 μg/ml of each dye. Apoptosis control was induced by 5 μM of H_2_O_2_ added to cell culture during 2 h. A Nikon Elipse 400 epifluorescence microscope was used and samples were analyzed by FITC/TRITC using the 20X or 40X Fluor objectives.

### Caspase-Glo-3/7 assays

The caspase-Glo-3/7 assay is a homogenous, luminescent assay that measures caspase-3 and caspase-7 activities. Briefly, a total of 2X10^4^ cells per well were plated in a 96-well plate. Caspase-Glo-3/7 reagent (Promega, Madison WI) containing 100 μl of blank reaction, negative control cells and treated cells in culture medium was added to each well of a white-walled 96-well plate. The blank reaction was used to measure background luminescence associated with the cell culture system and caspase-Glo-3/7 reagent. The plate was covered with the lid and gently mixed at 300 rpm for 30 s. The plate was incubated at room temperature for 1 h. After incubation, the luciferase activity was measured and normalized using the Dual-Glo luciferase assay system (Promega, Madison WI) in Glomax Multidetection equipment according to the manufacturer’s instructions. Cell apoptosis rate was calculated as the subtracted value for the blank reaction from experimental values X 100. The luminescence value corresponds to relative light units. Each assay was carried out three separate times.

### Statistical analysis

All experiments were performed at least three times. The data were analyzed and X^2^ test was carried out to compare frequencies between the different experimental groups. *P*-values less than 0.01 were considered to be statistically significant and were indicated with an asterisk (*).

## Results

### siRNA expression plasmids for miR-21 induce silencing of human microRNA miR-21

**Fig. 1:**
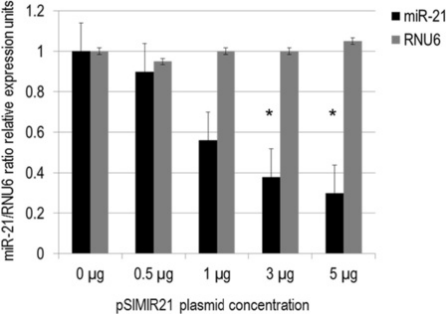
Silencing of human microRNA miR-21 expression by siRNAs. Quantitative real time RT-PCR analysis of miR-21 expression in SiHa cells transfected with pSIMIR21 plasmid. Total RNA and cDNA synthesis were obtained from 1 × 10^5^ SiHa cells (HPV16+) per well in a six-well plate containing DMEM at 37 °C with 5 % CO_2_ after 48 h transfection with pSIMIR21 plasmid (0, 0.5, 1, 3 and 5 μg). Relative expression by real-time RT-qPCR analysis of miR-21 was calculated using the 2^-∆∆Ct^ method and was normalized by miR-21/RNU6 ratio relative expression units. The Ct values were analyzed with pSilencer 1.0-U6 empty vector transfection and pSIMIR21 plasmid and values are presented as mean ± SD. The *P* values <0.01 are indicated with asterisks

The effect of siRNAs on miR-21 can be influenced by secondary structure and positioning of the cognate sequence within the pre-miR-21 molecule. To analyze the effect of the pSIMIR21 plasmid, we first determined whether siRNAs could induce specific silencing of miR-21 expression after transient transfection of pSIMIR21 plasmid. For this purpose, SiHa cells were transiently transfected with the pSIMIR21 plasmid and we analyzed the miR-21 expression level by real time RT-qPCR. As shown in Fig. 1, there was a significant decrease in the miR-21 transcript level when cells were transfected with pSIMIR21 plasmid at higher concentrations. After 48 h of transfection, the miR-21 expression level decreased by 70 % compared with cells transfected with pSilencer1.0-U6 plasmid (empty vector). We did not observe differences in miR-21 expression levels between SiHa cells treated with pSilencer1.0-U6 compared with non-transfected SiHa cells. The RNU6 RNA expression level did not show any changes under these same conditions. These data suggest that pSIMIR21 is a siRNA expression plasmid specific for miR-21, which has the ability to induce selective and specific silencing of miR-21 microRNA in human cervical cancer cells infected with HPV16.

### siRNA-mediated silencing of miR-21 expression has an effect on PTEN expression

**Fig. 2:**
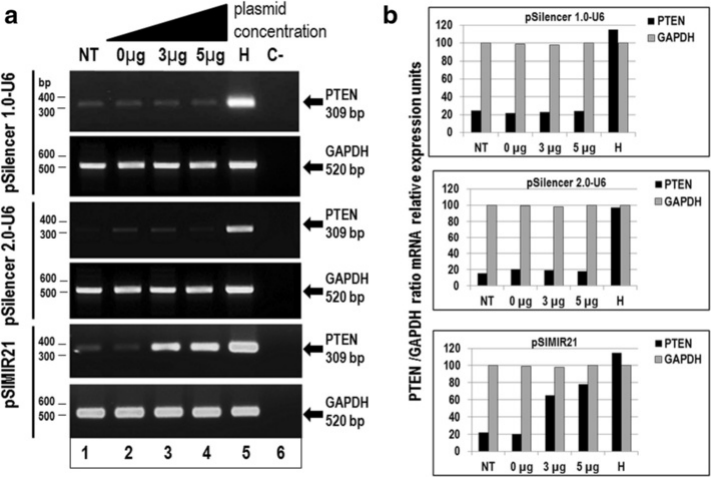
Analysis of PTEN gene expression by semiquantitative end-point RT-PCR after miR-21 silencing. Total RNA and cDNA synthesis were obtained from 1 × 10^5^ SiHa cells (HPV16+) per well in a six-well plate containing DMEM at 37 °C with 5 % CO_2_ after 48 h transfection with pSIMIR21 plasmid. Panel **a** Analysis of PTEN gene expression by semiquantitative end-point RT-PCR in SiHa cells non transfected (NT, lane 1) or transfected with pSilencer1.0-U6 plasmid or pSilencer2.0-U6 plasmid (empty vectors) or with pSIMIR21 plasmid (lanes 2 to 4). HaCat cell line was used as positive control (H, lane 5). PCR reaction without cDNA corresponds to reaction negative control (C-, lane 6). PCR amplification products were separated by electrophoresis in 1 % agarose gel. The DNA 100 bp ladder was used as molecular weight. Panel **b** PCR product bands were digitalized and analyzed by densitometer and data were analyzed by mRNA PTEN/mRNA GADPH ratio in relative expression units

In exploring miR-21 target genes, we focused on phosphatase and tensin homolog deleted on chromosome 10 (PTEN), a tumor suppressor gene whose protein product is involved in the removal of phosphate groups from key intracellular phosphoinositide 3-kinase signaling molecules. To achieve this aim, SiHa cells were transiently transfected with the pSIMIR21 siRNA expression plasmid to induce the silencing of miR-21, which is over expressed in this type of cells. With the goal of evaluating whether the PTEN gene is a cellular target of miR-21 in cervical cancer, we analyzed PTEN gene expression in SiHa cells transfected with pSIMIR21 plasmid, using end-point RT-PCR. As shown in Fig. 2, we found that siRNA against miR-21 has an effect on the expression of PTEN mRNA. Specifically, we found significant reestablishment of PTEN mRNA expression when cells were treated with siRNAs to miR-21 at a higher concentration. After 48 h of transfection with pSIMIR21, the PTEN expression level increased by more than 60 % compared with cells transfected with pSilencer 1.0-U6 (empty vector) as well as with the negative control pSilencer 2.0-U6 neo vector, which expresses a hairpin siRNA with limited homology to any know sequence in human genome. We did not observe differences in PTEN mRNA expression levels between SiHa cells transfected with pSilencer 1.0-U6 and negative control pSilencer 2.0-U6 neo vector, compared with untreated SiHa cells. The GAPDH mRNA expression level did not show any changes in similar transfection conditions. These data suggest that silencing of miR-21 microRNA can induce selective and specific reestablishment of PTEN gene expression in HPV16+ human cervical cancer cells.

**Fig. 3:**
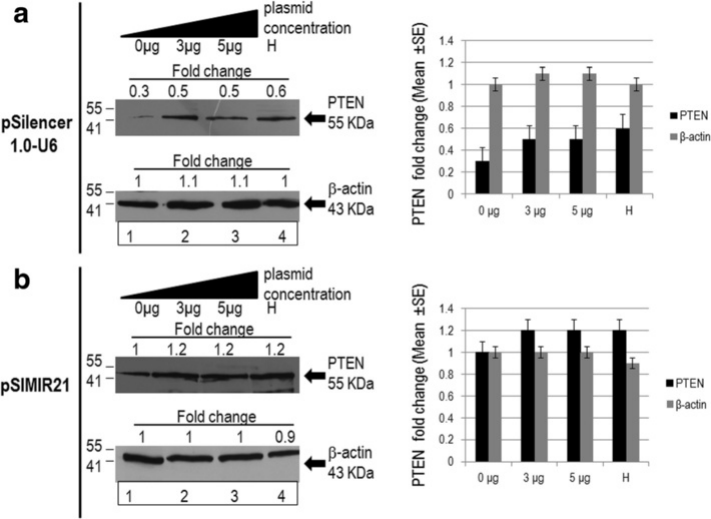
Analysis of PTEN protein expression by western blot after miR-21 silencing. Total cellular proteins were obtained from 1 × 10^5^ SiHa cells (HPV16+) per well in a six-well plate containing DMEM at 37 °C with 5 % CO_2_ after 48 h of transfection with pSilencer1.0-U6 plasmid or pSIMIR21 plasmid (lane 1 cells non transfected NT, lanes 2 and 3 cells transfected with the plasmids, lane 4 (H) corresponds to HaCat cells). The proteins were separated in 12 % SDS-PAGE and were transferred to nitrocellulose membranes which were incubated with each antibody. Panel **a** corresponds to transfection with pSilencer1.0-U6 plasmid. Panel **b** corresponds to transfection with pSIMIR21 plasmid and subsequent detection of PTEN protein. Amount similar proteins were analyzed in the immunoblots. The anti-beta-actin antibody was included as control

Furthermore, we analyzed whether the silencing effect of miR-21 alters PTEN protein expression. Using Western Blot assay, we identified the reestablishment of PTEN cellular protein expression after treatment of SiHa cells with siRNAs to miR-21 (Fig. 3). We used beta-actin protein as a control and did not observe any alteration in beta-actin cellular protein expression when SiHa cells were transfected with the pSIMIR21 plasmid. Thus, our results demonstrate that treatment of SiHa cells with siRNAs expressed in plasmid specific for miR-21 induces repression of miR-21 and reestablishment of PTEN gene expression and its protein product. Thus, expression of miR-21 microRNA is inversely correlated with PTEN expression, suggesting that PTEN is a miR-21 target gene in HPV16+ cervical tumor cells.

### Specific MRE recognition sequences by miR-21 are critical for regulation of PTEN

**Fig. 4:**
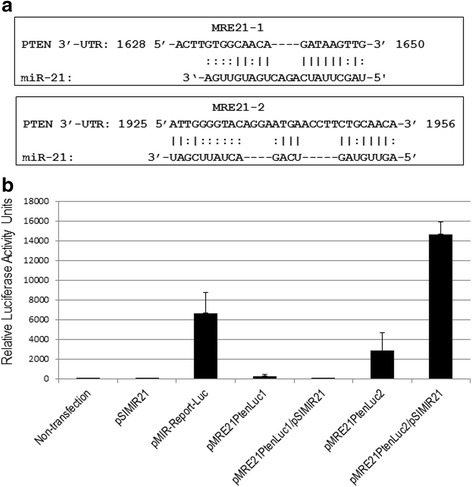
Functional analysis of MREs recognition sequences of PTEN gene by miR-21. Panel **a**. Nucleotide sequences of MRE21-1 and MRE21-2 of PTEN 3′-UTR regulatory region. MicroRNAs response elements of miR-21 (MRE21-1 of 1628 to 1650 nt and MRE21-2 of 1925 to 1965 nt) and complementary with miR-21 are indicated. Information was generated from nucleotide sequence database for PTEN [GeneBank: NM_000314.4] and for hsa-miR-21 [GeneBank: MI0000077]. Panel **b**. Regulation of PTEN 3′-UTR region modulated by miR-21 microRNA in SiHa cells non transfected (lane 1) or transfected with pSIMIR21 (lane 2), with pMIR-Report-Luciferase empty vector (lane 3), with pMRE21PtenLuc1 (lane 4), with pMRE21PtenLuc1 and pSIMIR21 (lane 5), with pMRE21PtenLuc2 (lane 6) and pMRE21PtenLuc2 and pSIMIR21 (lane 7) plasmids. After 48 h of transfection, luciferase activity levels were measured. Data shown represent the average of four independent experiments

In an effort to demonstrate that miR-21 directly targets the PTEN gene, two independent luciferase reporter plasmids were generated (pMRE21PtenLuc1 and pMRE21PtenLuc2), containing cloned microRNA response elements (MREs) to miR-21 from the PTEN 3′-UTR regulatory region (MRE21-1 of 1628 to 1650 nt and MRE21-2 of 1925 to 1956 nt) (Fig. 4a). SiHa cells were transiently transfected with pMRE21PtenLuc1 and pMRE21PtenLuc2 reporter plasmids independently to determinate the contribution of each MRE21 recognition site, and subsequently co-transfected with the pSIMIR21 plasmid to determine the effect of silencing miR-21. Transfection with pMRE21PtenLuc1 did not induce luciferase activity while transfection with pMRE21PtenLuc2 caused an approximately 60 % decrease in luciferase activity compared with SiHa cells transfected with pMIR-Report-Luciferase plasmid (empty vector). When SiHa cells were co-transfected with pSIMIR21 and pMRE21PtenLuc1 plasmids, the luciferase activity was very similar to control non-transfected SiHa cells. Interestingly, when SiHa cells were co-transfected with pSIMIR21 and pMRE21PtenLuc2 plasmids, luciferase activity increased twofold in comparison with transfection with pMIR-Report-Luciferase empty vector (Fig. 4b). These data suggest that MRE21-2 sequence is the main recognition site through which miR-21 mediates regulation of the PTEN gene. MRE21-2 is located from 1925 to 1956 nt downstream of the transcriptional start site of PTEN gene and appears particularly important for miR-21’s targeting mechanism of PTEN gene, given that MRE21-2 induced greater regulation of luciferase reporter gene. However, further investigation is needed to elucidate the specific molecular mechanism by which miR-21 interacts with MREs on the PTEN gene. These findings do suggest that a possible mechanism by which miR-21 regulates the PTEN gene in human cervical tumor cells is through interaction with the MRE21 recognition sites, principally MRE21-2.

### miR-21 silencing induces autophagy and apoptosis of cervical cancer cells

**Fig. 5:**
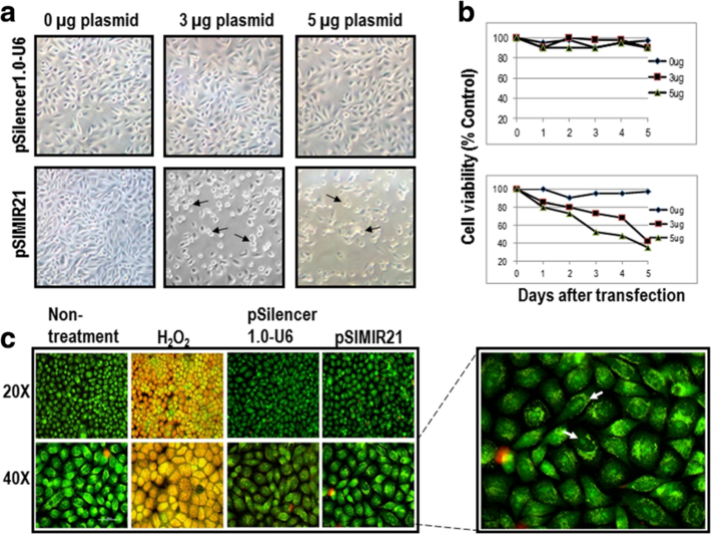
Analysis of tumor cell viability for silencing of miR-21 by siRNAs. Panel **a** SiHa cells were analyzed by white light microscopy (20X) 48 h after transfection with pSIMIR21 plasmid. The black arrows indicate the dead cells. Panel **b** Cellular viability was measured using MTS assay kit. Panel **c** SiHa cells attached on a slide were harvested at 48 h after transfection and stained with 5 μg/ml of acridine orange and propidium iodide dyes. Apoptosis control was induced by 5 μM of H_2_O_2_ added to cell culture during 2 h. A Nikon Elipse 400 epifluorescence microscope was used and samples were analyzed by FITC/TRITC using the 20X and 40X Fluor objectives. The white arrows indicate nucleus fragmented

To determine whether silencing of miR-21 expression by siRNAs affects cellular viability, MTS assays were carried-out on days 0, 1, 2, 3, 4 and 5 after transfection; using equal amounts of SiHa cells transfected with pSIMIR21 plasmid. Silencing of miR-21 decreased the viability of SiHa cells compared with cells transfected with pSilencer 1.0-U6 empty vector (Fig. 5a, b). A marked decrease in cellular viability was observed from days 2 to 5 after treatment with siRNAs to miR-21.

**Fig. 6:**
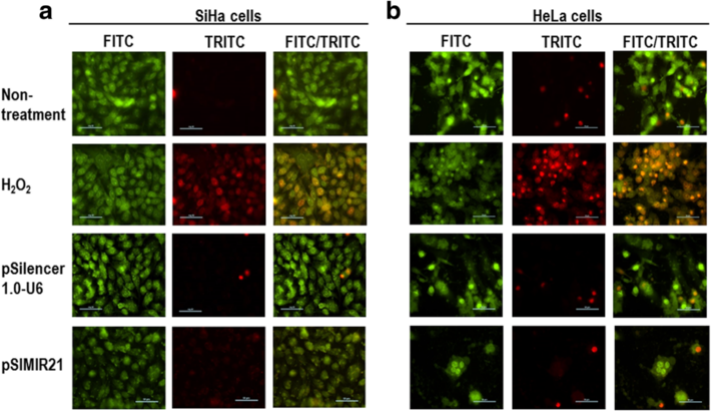
Epifluorescence staining with Acridine Orange (AO) and Propidium Iodide (PI) in response to miR-21 silencing. SiHa (panel **a**) and HeLa cells (panel **b**) were plated at 70 % confluent on glass coverslips. Attached samples of SiHa and HeLa cells on a slide were harvested at 48 h after transfection with pSilencer 1.0-U6 or pSIMIR21 plasmids and stained with 5 μg/ml of AO and PI dyes. Apoptosis control was induced by 5 μM of H_2_O_2_ added to cell culture during 2 h. Following transfection, cells were observed in a Nikon Elipse 400 epifluorescence microscopy and samples were analyzed by FITC/TRITC using the 40X Fluor objective

The staining with acridine orange of SiHa cells (Fig. 5c) and HeLa non-treatment control cells showed predominantly green fluorescence with minimal red fluorescence in cytoplasmic and nuclear components (Fig. 6). The cells transfected with pSilencer 1.0-U6 empty vector showed red fluorescence, and cells in which miR-21 was silenced displayed considerable red fluorescence, suggesting the formation of numerous acidic autophagolysosomal vacuoles and induction of early apoptosis. As seen in Fig. 6a, 48 h post-treatment with pSIMIR21, SiHa cells presented a typical morphology of early apoptotic cells with fragmented nucleus and compromised nuclear membrane, similar to the positive control of apoptosis in treatment with H_2_O_2_. This effect was not observed in non-transfected control cells or cells transfected with empty vector. In HeLa cells 48 h post-treatment with pSIMIR21, the quantity of cells was lower compared to SiHa cells, suggesting that these cells were more sensitive to the treatment with silencing of miR-21 (Fig. 6b). In fact, HeLa cells presented a typical morphology of early apoptotic cells with fragmented nuclei but necrosis was also observed. The experimental cells differ from the positive apoptosis control treated with H_2_O_2_ in that compromise of the nuclear membrane and chromatin condensation were observed. Control untreated cells and cells transfected with pSilencer 1.0-U6 empty vector were not affected and showed a viable morphology.

**Fig. 7:**
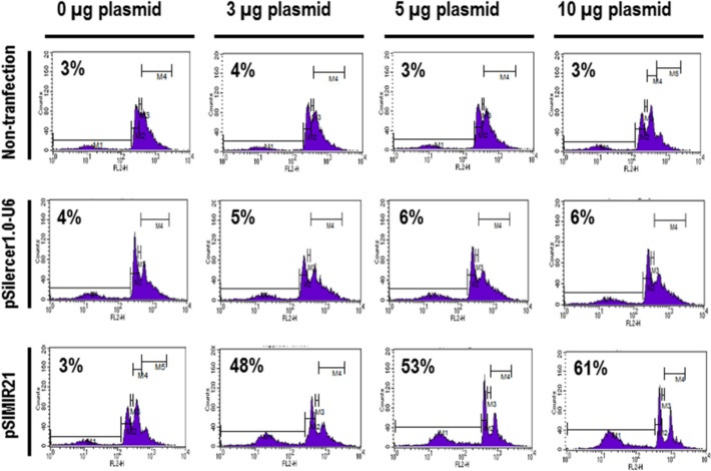
Apoptosis analysis of cervical cancer cells by silencing of miR-21. SiHa cells were non-transfected or transfected with pSilence1.0-U6 or pSIMIR21 plasmids and were harvested and fixed in 70 % ethanol. Cells were treated with propidium iodide and approximately 100,000 nuclei were processed by FASC-Sort. The assays were normalized with non-transfected SiHa cells and the data were analyzed with ModFitLT software

The ideal therapeutic agent in cancer treatment would selectively induce death of tumor cells without affecting surrounding normal cells. However, the biological effect of silencing miR-21 in cervical cancer cells is not well understood. Therefore, we analyzed the effect of silencing miR-21 gene expression, using siRNAs against miR-21, on survival of SiHa cells. Analysis of cellular DNA content by flow cytometry at different phases of the cell cycle is an effective method to determine whether cells are proliferating or going through cell death by the process of apoptosis. We assessed DNA content using flow cytometry analysis in SiHa cells transiently transfected with the pSIMIR21 plasmid. After 48 h of transfection, cell death by apoptosis occurred in 53 to 61 % in SiHa cells transfected with the pSIMIR21 plasmid, while 3 to 6 % of SiHa cells underwent cell death when they were not-transfected or transfected with the pSilencer 1.0-U6 empty vector (Fig. 7). These findings suggest that induction of cell death by autophagy and apoptosis in human cervical cells transfected with pSIMIR21, which produces specific siRNAs toward miR-21, is mediated by silencing of this microRNA’s expression and is selective to cancer cells transformed with HPV16 and HPV18.

### Caspase activation in response to miR-21 silencing

**Fig. 8:**
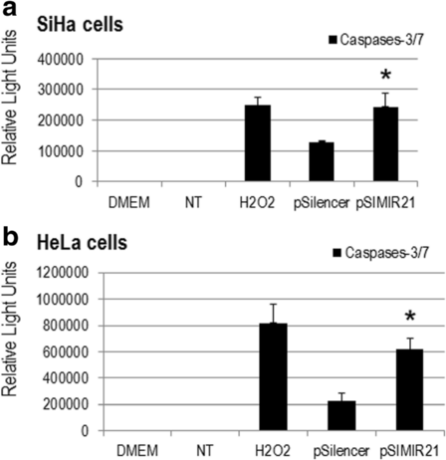
Detection of caspases activity in response to miR-21 silencing. SiHa (panel **a**) and HeLa (panel **b**) cells were analyzed by caspase-3/7 activities, 48 h after of non-treated (lane NT), treated with H_2_O_2_ (apoptosis control), transfected with empty vector (pSilencer 1.0-U6) and transfected with pSIMIR21 plasmids. DMEM medium alone was included. Following transfection, luciferase activity levels were measured and luminescence value corresponded to relative light units. The values are presented as mean ± SD and *P* values <0.01 are indicated with asterisks. Each assay was carried out three separate times

The activity of caspases 3 and 7 can be measured by the caspase-Glo-3/7 assay. Their activity generates a luminogenic caspase-3/7 substrate, which contains the tetrapeptide sequence DEVD-AMC, in a reagent optimized for caspase activity, luciferase activity and cell lysis. The addition of a single caspase-Glo-3/7 reagent in an add-mix-measure format results in cell lysis, followed by caspase cleavage of the substrate and generation of a glow-type luminescent signal, produced by luciferase. Luminescence is proportional to the amount of caspase activity present. In assays carried out in parallel, the activity of caspase-3 and caspase-7 were determined together because caspase-3 and −7 utilizes the same substrate DEVD-AMC; therefore, the activity determined by cleavage of DEVD-AMC is in fact contributed by both of these caspases. In Fig. 8 we demonstrate that silencing of miR-21 in SiHa and HeLa cells induced a significant increase in activity of caspase-3/7 compared with cells transfected with pSilencer 1.0-U6 empty vector. Cells treated with H_2_O_2_ were used as positive apoptotic controls. We did not observe significant differences in caspase-3/7 activity levels between untreated SiHa cells and DMEM media alone. Therefore, these data suggest that silencing miR-21 has implications on the apoptosis pathway mediated by caspase-3/7 and results in induction of early apoptosis in cervical cancer cells.

Taken together, these evidences indicate that administration of siRNAs expressed in plasmid against miR-21 can induce silencing of miR-21 gene expression and reestablish PTEN gene and protein expression, confirming that PTEN is a target gene regulated by miR-21 through MRE21-2. Thus, miR-21 appears to have biological effects on human cervical cancer cells transformed by HPV16 through the regulation of cell death by autophagy and apoptosis.

## Discussion

In the present study we demonstrate that in cervical cancer, the overexpression of miR-21 can contribute to the carcinogenesis process by altering the expression of cellular genes involved in checkpoint regulation, including PTEN, which inhibits tumor progression. Here we report that miR-21 post-transcriptionally down-regulates the expression of PTEN and inhibits cell proliferation and survival of cervical cancer cells. MiR-21 is the most abundantly expressed microRNA in cervical cancer cell lines as well as in tumor samples [32–34]; its overexpression in some HPV-associated cervical cancers may be related to the integration of HPV16 at fragile site FRA17B [46], the region in which the gene locus of miR-21 is located. While the genes and downstream pathways targeted by miR-21 remain in large part to be elucidated, the tumor suppressor gene PTEN has been validated as a miR-21 target in hepatocellular cancer [7], cholangiocarcinoma [8], non-small cell lung cancer [28] and recently in cervical cancer [55, 56, 64]. Inhibition of expression of PTEN can cause cellular transformation and neoplastic growth by eliminating the “off switch” for cellular growth and survival signals produced by the PI3K/Akt pathway [47]. In human gastric cancer, knockdown of miR-21 increased expression of PTEN and decreased tumor cell proliferation, migration and invasion [48].

We observed that PTEN gene expression and PTEN cell protein expression were reestablished in HPV16+ human cervical cancer cells when miR-21 expression was silenced with specific siRNAs expressed in plasmids (Figs. 2 and 3). A previous study reported the disruption of miR-21 by genome editing using transcription activator-like effector nucleases (TALENs) in cancer cells [49]. In the study, by analyzing single cell-derived miR-21 knockout clones, it was determined that HeLa cells (HPV18 positive) lacking miR-21 were phenotypically less malignantly transformed and more sensitive to cisplatin. Furthermore, the loss of miR-21 led to a subtle global increase in mRNAs containing miR-21 target sequences. Interestingly, a slight increase in PTEN protein expression in the miR-21 knockout was observed. These findings using TALEN-mediated microRNA gene disruption in HeLa cells are consistent with our data reported here for SiHa cells (HPV16 positive) using siRNA expression plasmids, and demonstrate the value of knockdown using both the techniques of siRNAs and TALEN in studies of human carcinogenesis. Given that currently more than 30 miRNAs have been validated to target PTEN [50], the depletion or silencing of miR-21 may not on its own explain a drastic decrease in PTEN expression (Fig. 3). This data suggests that PTEN gene expression is modulated at the post-transcriptional level by miR-21, and its regulation is mainly through miR-21’s interaction with microRNA response elements (MREs) localized in the 3′-UTR regulatory region of the human PTEN gene (Fig. 4). This evidence is relevant in the context of reports that PTEN regulates several cellular checkpoints including the processes of apoptosis [51], growth, invasion, migration [52] and angiogenesis [53] through its effects on several signaling pathways. PTEN functions as a dual specificity phospahatase and its key target is phosphatidylinositol 3, 4, 5,-triphosphate, the direct product of the phosphatidylinositol 3-kinase (PI3K). Thus, the PTEN/PI3K/AKT circuit is a signal pathway implicated in numerous carcinogenesis-related processes.

Although multiple mechanisms such as genetic mutation, promoter methylation and post-transcriptional modification may contribute to deregulation of the PTEN gene, two microRNA response elements (MRE21-1 and MRE21-2) have been reported to be involved in PTEN regulation in several cancers [28, 54]. MREs are binding sequences in the 3′-UTR of mRNA through which microRNAs suppress their target gene. PTEN MRE sequences for miR-21 have been reported in the online databases miRanda, Target Scan Human and miRBase, and several groups have identified and evaluated the putative MRE sequences in PTEN’s 3′-UTR. MRE21-1 was identified and evaluated in lung cancer cells; it corresponds to a 22-nucleotide sequence from nucleotide 1628 to 1650 in the PTEN 3′-UTR [28]. While MRE21-2 was identified and analyzed in glioblastoma cells and corresponds to a 31-nucleotide sequence from nucleotide 1925 to 1956 in the PTEN 3′-UTR [54]. Interestingly, we observed that MRE21-2 was the principal regulatory target of miR-21 in human cervical cancer cells transformed with HPV16, which we confirmed by silencing miR-21 (Fig. 4). This finding suggests that the MREs within the same gene can induce different responses depending on the biological context and regulatory genetic networks, which helps to explain in part why one microRNA can function as an oncomir in a particular cancer and as a tumor suppressor gene in a different malignancy.

On the other hand, another study assessed the functional contribution of Let-7a and miR-21, which interact with STAT3 signaling and/or its downstream effects in HPV16-positive cervical cancer cells [55]. The specific targeting of miR-21 using a miR-21 inhibitor resulted in an increased level of PTEN expression, thus negatively regulating STAT3. In addition, siRNA-mediated inhibition of HPV E6 oncoprotein resulted in loss of miR-21, reduction in STAT3 and increase in PTEN. It is likely that STAT3-induced miR-21 contributes to a relevant part of positive feedback loop in cervical cancer cells that keeps several apoptosis-inducing regulators, including PTEN, under control, and that miR-21 inhibition relieves PTEN suppression leading to inhibition of STAT3 signaling. In addition, this same group reported clinical evidence of the existence of a functional loop involving miR-21 and Let-7a as regulators of STAT3 signaling in different grades of cervical tissues derived from pre-cancerous and cancerous lesions (LSIL and HSIL) which represent the natural history of HPV-induced cervical carcinogenesis [56]. The data revealed overexpression of miR-21 in pre-cancer and cervical cancer lesions; the level increased from LSIL to HSIL. In this study, a high level of PTEN protein was detected in normal cervical tissues, while it was diminished in tissues from pre-cancerous and cancerous lesions.

Taken together, these findings suggest the existence of a functional circuit involving Let-7a, STAT3, miR-21 and PTEN in cervical cancer cells. This functional circuit comprises the regulatory genetic network of miR-21 in a positive feedback loop through which down-regulation of PTEN keeps STAT3 activity under control, as reported previously [57]. Our data reported here demonstrate that miR-21 targets its regulator PTEN, forming a self-adapting feedback loop and creating a balance mechanism. This supports the existence of this regulatory genetic network in cervical cancer cells. Our data in cervical cancer cell lines and the evidence in tumor tissues suggest that the miR-21/Let-7a/STAT3/PTEN regulatory network may be able to be used as a biomarker in cervical cancer; however, it is important to be cautious because its expression can be highly variable within cervical tissues in different subpopulations. Taken together, these findings support the notion that microRNAs function cooperatively with transcription factors in regulating a set of target genes, allowing coordinated modulation of gene expression, both transcriptionally and post-transcriptionally. Thus, our results could contribute to the identification of more core factors, other parallel regulatory genetic networks and relevant motifs in cervical carcinogenesis and/or other tumorigenesis processes.

The regulatory genetic networks involved in cervical cancer, including microRNAs, target genes, transcription factors and host genes, have been increasingly better characterized [57]. One sub-network centering on PTEN as its principal component has been described. According to the regulatory genetic network data, miR-21 and PTEN were found to be differentially expressed in cancer cells; together with miR-214, they form an ordered control system. This regulatory genetic network has a self-adaptation feedback loop providing a balance mechanism for maintenance of cell homeostasis. Thus, the circling between miR-21 and PTEN as dominant and dominating factors simultaneously may explain the biological effect of inhibition of cell proliferation induced by silencing miR-21 and reestablishing PTEN expression.

Another relevant regulatory genetic network is mediated by p53. Several microRNAs have been reported to be involved in the p53 pathway, either being regulated by p53 or acting directly to suppress the expression of p53 or its downstream effectors, suggesting an important role for microRNAs in the p53 signaling pathway [58–61]. There is no known miR-21 response element in the p53 3′-UTR in either human or mouse to date. However, several studies have reported that miR-21 suppresses the expression of multiple genes in the p53 network. Downregulation of miR-21 in glioblastoma cells leads to increased expression of p53 and activators of the p53 pathway, including JMY, TOPORS, TP53BPS and DAXX, leading to cell cycle arrest and apoptosis [27]. In breast cancer cells, inhibition of miR-21 induces the expression of several genes regulated by p53 at the mRNA level, including FAM3C, ACTA2, APAF1, BTC2, FAS, CDKN1A (p21), and SESN1 [62]. These genes are required for p53 function, suggesting that miR-21 overexpression may impair the tumor-suppressive function of the p53 pathway. A recent study reports that miR-21 expression is elevated in human lung tumors with mutated p53 and distant metastases, and that increased expression of miR-21 confers increased invasiveness in *Trp53*-deficient mouse tumors [63]. However, the functional interaction between p53 and miR-21 in vivo has remained elusive. Given the existing evidence, it is certainly conceivable that miR-21 regulates the expression of multiple cellular genes through PTEN, p53 and associated regulatory genetic networks, which act as checkpoints to maintain cell homeostasis. The miR-21-PTEN ordered control system has a critical and more complex function than originally thought, representing a regulatory genetic network highly involved in the processes of carcinogenesis.

We also sought to determine whether the silencing of miR-21 by siRNAs has an effect on cancer cell proliferation. We found that siRNAs can silence miR-21 gene expression, causing a decrease in the level of miR-21 mRNA expression in human cervical cancer cells. To elucidate the mechanism by which the silencing of miR-21 inhibits the growth of SiHa cells, we performed cell viability evaluations (Fig. 5) and induction of cellular death (Figs. 6 and 7). We observed inhibition of SiHa cell proliferation and induction of autophagy and apoptosis. In addition, the upregulation of miR-21 in invasive cervical cancer tissues has been reported, and studies have confirmed that miR-21 promotes proliferation, migration and invasion in CaSki (HPV16-positive) or HeLa cells, by downregulating the expression of tumor-repressive PTEN [64]. This effect can be explained by the fact that PTEN inhibits cell migration and invasion by directly dephosphorylating two key tyrosine-phosphorylated proteins, thereby antagonizing the interaction of integrins with the extracellular matrix and integrin-triggered signaling pathways [65]. The dephosphorylating function of PTEN is also necessary in the lipid signal transduction PI3K pathway [53]. Therefore, PTEN acts as a unique tumor suppressor by inhibiting lipid phosphatase and protein tyrosine phosphatase activity, thus negatively regulating cell proliferation and invasion. Our results are in accordance with this scenario because we report that overexpression of miR-21 post-transcriptionally downregulates the expression of PTEN, inhibits cell proliferation and promotes survival of SiHa and HeLa cells, which are human cervical cancer cells transformed with HPV16 and HPV18, respectively. The principal novel contribution of our data is to demonstrate that PTEN gene expression is regulated by miR-21 and this regulation occurs mainly through MRE21-2 within the PTEN 3′-UTR in cervical cancer cells. This evidence strongly supports the notion of a regulatory genetic network between miR-21 and PTEN in cervical cancer cells.

During autophagy, acidic autophagic vacuoles, also called autophagolysosomes, are formed as a result of fusion of autophagosomes with lysosomes [66]. These are a characteristic feature of cells engaged in autophagy. Formation of autophagolysosomes can be detected by fluorescence microscopy after staining with the lysomotropic agent acridine orange. Staining of untreated cells with acridine orange, a weak base generates green fluorescence of the cytoplasmic and nuclear components. In larger acidic compartments such as autophagolysosomes, the protonated form of acridine orange accumulates and generates red fluorescence, when can be observed by fluorescence microscopy (Fig. 5c). We analyzed the effect of silencing miR-21 on autophagy and early apoptosis by looking at formation of autophagolysosomes with fluorescence microscopy after staining with acridine orange and propidium iodide (Fig. 6). The results of both the fluorescence microscopy and western blot assays indicate that silencing of miR-21 leads to increased PTEN gene expression and are associated with autophagy and early apoptosis processes. These mechanisms of programmed cell death have been described previously in renal tubular epithelial cells, and play an important role in cell survival in response to stress [67].

We demonstrated that the processes of autophagy and apoptosis occur in response to silencing of miR-21 and are required for adaptation to conditions of stress in cervical cancer cells. Thus, the induction of autophagy during silencing of miR-21 in cervical cancer cells may provide for an appropriate environment to maintain cellular homeostasis before reaching the threshold for apoptosis. Therefore, this evidence supports and strengthens the concept of a regulatory genetic network involving miR-21 in a positive feedback loop and down-regulation of PTEN in human cervical cancer cells.

The ability to selectively silence mammalian gene expression using siRNAs offers new and exciting perspectives in the field of mammalian cell biology and pathology. However, it cannot be assumed that all genes will be equally susceptible to the siRNA mechanism. SiRNAs have been shown to knock down a large number of genes expressed in mammalian cells and siRNA technology has been used to characterize mammalian gene function [68–70]. An application of the siRNAs with great potential is in specific gene therapy against cancer. SiRNAs mimic endogenous microRNAs, which can effectively inhibit the translation of target mRNAs by binding the mRNA 3′-UTR. This process is dependent upon mRNA accessibility and, within the target mRNA molecule, accessibility of the short internal nucleotide sequences that are homologous to the siRNA transcripts. Therefore, various factors can influence the susceptibility of a given mRNA transcript to siRNA-mediated degradation. These include mRNA secondary structures and proteins which package mRNA for translocation within the cell. Other protein-mRNA interactions are also relevant which direct a given mRNA to a specific sub-cellular locus, and those mRNAs which can be bound by the proteins they encode.

In summary, the findings of our investigation demonstrate that siRNAs against miR-21 can produce a specific decrease of miR-21 mRNA expression and reestablish PTEN gene and protein expression, as well as lead to growth suppression and induction of cell death by autophagy and apoptosis. Thus, the biological effects of miR-21 in cervical cancer cells can be explained, at least in part, by miR-21’s regulation of the PTEN pathway. The main novel contribution of our data is to demonstrate that in cervical cancer, PTEN gene expression is regulated by miR-21 and this regulation is mediated mainly through the MRE21-2 recognition sequence within PTEN’s 3′-UTR. We also demonstrate that silencing miR-21 induces the death of cervical cancer cells. This evidence strongly supports the existence and relevance of a regulatory genetic network between miR-21 and PTEN in cervical cancer cells.

## Conclusions

The findings and implications of this study are as follows: i) The administration of siRNAs expressed in plasmids specific to microRNA miR-21 induces the silencing of miR-21 in human cervical cancer cells transformed by HPV16. ii) The silencing of miR-21 induces reestablishment of PTEN gene and protein expression. iii) The reestablishment of PTEN may explain the biological effects of silencing miR-21, which include induction of cell death by autophagy and apoptosis in cervical cancer cells. iv) The silencing of miR-21 expression by siRNAs and its effects upon PTEN does not exclude other potential molecular pathways in cervical carcinogenesis that may be influenced by the silencing of miR-21, and there exist excellent opportunities to identify new therapeutic molecular targets. Our findings suggest that a therapeutic strategy employing siRNAs could effectively inhibit the growth of virus-related cancers. The clinical relevance of silencing miR-21 expression with siRNAs will be better appreciated once they are applied in clinical trial protocols. This goal will require selection of adequate cloning vectors for siRNAs, development of molecular vehicles to administer siRNAs at the tumor site, and design of treatment schemes such as chemotherapy, radiotherapy or immunotherapy treatment to be used in combination with siRNAs. Our results indicate that siRNAs have a great potential in the treatment of human malignancies such as cervical cancer. There is no doubt that the siRNA technology platform is a realistic gene therapy strategy against the development of cervical cancer. The challenge is now to develop efficient strategies for the application of this technology in clinical trials.

## Abbreviations

3′-UTRs: 3′-untranslated regions
HNRPK: heterogeneous nuclear ribonucleoprotein K
HPV: human papillomavirus
miR-21: microRNA-21
MREs: microRNA response elements
pri-miR-21: primary transcript containing miR-21
PTEN: phosphatase and tensin homologue deleted on chromosome ten
RECK: reversion-inducing-cysteine-rich protein with kazal motifs
siRNAs: small interference RNAs
TPM1: tropomyosin 1
